# Insertion-deletion polymorphisms (indels) as genetic markers in natural populations

**DOI:** 10.1186/1471-2156-9-8

**Published:** 2008-01-22

**Authors:** Ülo Väli, Mikael Brandström, Malin Johansson, Hans Ellegren

**Affiliations:** 1 Department of Evolutionary Biology, Evolutionary Biology Centre, Uppsala University, Uppsala, Sweden; 2 Institute of Agricultural and Environmental Sciences, Estonian University of Life Sciences, Tartu, Estonia

## Abstract

**Background:**

We introduce the use of short insertion-deletion polymorphisms (indels) for genetic analysis of natural populations.

**Results:**

Sequence reads from light shot-gun sequencing efforts of different dog breeds were aligned to the dog genome reference sequence and gaps corresponding to indels were identified. One hundred candidate markers (4-bp indels) were selected and genotyped in unrelated dogs (n = 7) and wolves (n = 18). Eighty-one and 76 out of 94 could be validated as polymorphic loci in the respective sample. Mean indel heterozygosity in a diverse set of wolves was 19%, and 74% of the loci had a minor allele frequency of >10%. Indels found to be polymorphic in wolves were subsequently genotyped in a highly bottlenecked Scandinavian wolf population. Fifty-one loci turned out to be polymorphic, showing their utility even in a population with low genetic diversity. In this population, individual heterozygosity measured at indel and microsatellite loci were highly correlated.

**Conclusion:**

With an increasing amount of sequence information gathered from non-model organisms, we suggest that indels will come to form an important source of genetic markers, easy and cheap to genotype, for studies of natural populations.

## Background

Advancement in population and evolutionary genetic research has been accompanied by – or perhaps better phrased – been a consequence of continuous improvement in the way genetic similarity or dissimilarity between genomes is assessed. Seen in long time perspective, genetic marker methodology has evolved from focusing on phenotypes, via immunological parameters and proteins, to genotypes. Following their introduction to the study of natural populations about 15 years ago [1-3], microsatellites or short simple tandem repeats have been the genotype-based marker approach of choice for many applications where the relatedness between individuals, populations or species is sought. Preceding and subsequently in parallel to this, non-repetitive DNA sequence variation has been assessed through various approaches, including DNA sequencing, restriction fragment length polymorphism (RFLP) analysis, single strand conformation polymorphism (SSCP) analysis, random amplified polymorphism detection (RAPD) and amplified fragment length polymorphism (AFLP) analysis [4]. More recently, single nucleotide polymorphisms (SNPs) are increasingly finding their application in studies of natural populations [5,6].

The benefits of microsatellites are several and well-known. They are multi-allelic, show high heterozygosity and are relatively easy to analyse at moderate cost. Because of the high polymorphism information content, a rather limited number of markers suffice for many applications in molecular ecology and population genetics. It is usually not too difficult to isolate the required markers from DNA libraries [7] or to employ markers originally developed for related species [8]. SNPs merit as genetic markers for other reasons. They are very common, with genomic densities outnumbering that of microsatellites by orders of magnitudes. Large numbers of individuals may be genotyped at large number of loci by simple and fast automatic methods, and data interpretation is usually straightforward [5,9]. Moreover, SNP variation at protein-coding genes and in other functionally constrained regions of the genome is likely to form the main genetic background to phenotypic variation. Furthermore biallelic SNPs evolve in a manner well described by simple mutation models. There are good reasons to believe that they in many cases will gradually come to replace the use microsatellites in molecular ecology and population genetics/genomics research [6].

Unfortunately, however useful, both microsatellites and SNPs suffer from some shortcomings. The complex and heterogenous mutation pattern of microsatellites [10] introduces ambiguities to further data analysis. Genotyping errors may occur because of stutter bands and technical artefacts (allelic dropouts, null alleles, false alleles, size homoplasy) [11]. As for SNPs, many more markers are needed to get the same amount of information [6,9]. Moreover, despite the many elegant genotyping methods available [9], most of them are relatively costly at small or medium scales, and requires special equipment for high-throughput genotyping.

With a few years' lag phase, the introduction of new genetic markers to the study of natural populations has generally followed methodological developments made in the genetic analysis of model organisms [4]. Currently, there is an increasing focus on polymorphisms of the type short insertions and deletions (indels) in genomic research of humans [12,13] and model species such as *Drosophila melanogaster *[14] and chicken *G. gallus *[15]. Indels have been recognised as an abundant source of genetic markers that are widely spread across the genome, though not as common as SNPs. For instance, Mills et al. [13] used data from re-sequencing surveys to identify 415,436 indels segregating in human populations and they estimated that among the total number of >10 million polymorphisms known in humans, some 1.5 million represent indels. Clearly, this indicates that indels could form a very common class of genetic markers also in non-model species and this is particularly so given that genetic diversity in many natural populations typically seems to be higher than in humans [5,6,16]. Most importantly, indels can be genotyped with simple procedures based on size separation. Another advantage is the minuscule chance of two indel mutations of exactly the same length happening at the same genomic position, meaning that shared indels can confidently been seen as representing identity-by-descent [cf. [17]].

In this study we present a test of the usefulness of indel markers in natural populations. We use a bioinformatics approach to survey dog shot-gun reads [18] for the presence of indels and based on this we design a pipeline for development of PCR-based indel markers. We subsequently genotype 100 indels in natural wolf populations and compare the results with data on microsatellite variability obtained from the same animals.

## Results

There are ≈100,000 shot-gun reads available from each of 9 different dog breeds, sequences data that come in addition to data obtained for the partial [19] or full genome sequencing [18] of two dogs. We surveyed 200,000 of these trace reads for the occurrence of short insertion and deletion polymorphisms, as detected by alignment against the reference sequence of one female boxer [18]. Note that there is essentially no sequence overlap among trace reads so the alignments were consistently in the form of only two alleles drawn from the population of dogs. In total, this yielded 30,116 length polymorphisms, corresponding to about one length variant every 2400 bp. Consistent with what has been found in other organisms cf. [12,13,15,20], the great majority of indels were very short with a dominance of 1-bp events (Table 1). From these polymorphism data we chose 4-bp indels for further analysis since they are easily scored by size separation and relatively abundant in the genome. We selected 100 4-bp non-repetitive indels located within unique sequence. They were spread across the canine genome and consistently represented autosomal loci; the great majority of them likely to reside in non-protein coding sequence. Of the 100, 94 could be readily amplified and scored and were selected for further analysis (Table 2).

**Table 1:** Number and density of indels found.

Indel size (bp)	Count	Density (indels per million bp)
1	20558	284.6
2	3352	46.4
3	1942	26.9
4	2185	30.2
5	678	9.4
6	436	6.0
7	297	4.1
8	297	4.1
9	219	3.0
10	152	2.1

**Table 2:** Location of selected indel-markers (position on the respective chromosome in the dog genome assembly), primer sequences, amplicon length and expected heterozygosities from the genotyping of 18 wolves from worldwide.

**No.**	**Chromosome**	**Position**	**Primer F**	**Primer R**	**Fragment length (bp)**	**He**
1	23	35377868	ccaggcttgtgtgaagctct	gccttgttggtttcagtggt	126	0.43
2	21	44954960	tgtcatttggccagatctctaa	ttggattaaccctaccacacg	117	0.26
3	5	56432744	catgctgcttgaagtgcaata	tctctgtgtgcctctcatgaat	123	0.00
4	6	31824728	cacaatgaccacttattaaagattaca	ctaggatgagagcccagctt	139	0.00
5	29	17604384	tgtcaggtttcatatccttttgtg	gaactatccttaaatagaaccaatgc	111	0.29
6	15	52623379	ttcacatccatctgtcttgga	acccgggagtttgcctatac	125	0.20
7	12	4364137	ctcctgttccctccagca	ggaccatgctgtggatctg	115	0.17
8	30	32665884	agaccagggtctgaatttgc	tttccaaggtcccaccacta	116	0.24
9	7	52754426	ttcacaaattgctatacctaaaaatg	ttcctgtgggcataataatca	136	0.48
10	26	32666193	tccaagaacaaagaagtaatgtaaaa	ggaattgatttactgatagtgagatg	130	0.39
11	37	18098142	gaaaggtccctctgaattgaa	tctgtgctcttcactggaaaaa	122	0.18
12	14	38060282	gtgtgctctaggggccatt	gaatgaaatcatggaagagcaa	115	0.30
13	17	46184901	gaagggacaaaaccttggaa	tgaactaccctcgtgatcca	139	0.37
14	28	38785954	aaaggagggcttgcagtttt	ttctccttttagaccctttgtca	90	0.32
15	25	8846761	tgccttagcgttggcatt	tggtgctctttcttgttgga	169	0.36
16	34	7543229	caggagcaaagtaagggtaatca	gctttggtattgttgattctattgtaa	90	0.44
17	20	21505420	aatggggacaccagtcactt	tgggagttctggctccac	139	0.00
18	1	32746664	tcctgcggcagtttgg	ccaagattgtgcatgtcagg	104	0.36
19	23	42171234	caaaggcaagaaggcagatg	tacatggtccctgtgttcca	81	0.51
20	35	25523850	ttagcgatgttgagcgttttt	ttcccttaagaaataggcagagg	92	Excluded
21	22	41896467	tgcaaaggagtgggaattatc	tggatgttaaaaacctggtatattgt	164	0.37
22	30	32665884	ccaagccccttccaatacta	tttccaaggtcccaccacta	92	0.29
23	10	55417044	tgctttgcatgttacattcttca	catgtcatagtcacatgctgtacg	151	0.32
24	22	20849044	tattgctgccctgtttcaga	gctgaaggaaatatctgttgaatg	102	0.16
25	24	40849741	ctgcggtctcacatccttag	tgagggggatttgatctctt	69	0.14
26	31	8148287	tctgctcaggtttagccttg	ggatgcaagaaaatctgctg	80	0.43
27	3	67261495	ttactcccagctctgtgcat	gacccaggtggggatatcta	88	0.06
28	26	37097342	tgcccccactactcttgc	aaagggtgatggtcctttga	98	0.09
29	1	109880549	tgttgagcccttgaaatgag	agcgaaaagtggcagtgg	68	0.51
30	13	20047698	tggctgccccatcttatg	cacaatggcagaacacgag	78	0.06
31	10	13999933	gccttcttcctctgcctct	ctcaggcaggcaaataaaaa	90	0.13
32	5	6092689	gcttgggaaatcatggtca	gctaaggaaagcaagctgga	100	0.50
33	3	52499600	tctgactggcctccttcg	attcaagtgtgcccgagag	67	0.51
34	2	87005110	gccgccgtgtcttgtc	cgaatgcgtgcttaccg	80	0.07
35	5	16794632	cgatgctggtgaggaagc	ccatccctgagccacct	91	Excluded
36	1	40637985	aagggccgatgccagt	caggttcttgtttccccaaa	100	0.42
37	14	16593885	cccaggtgccccttattt	ggctcatgctgctctgg	110	0.00
38	38	3128143	gcttcccttgtttctttcca	tgcccatgtaccaaatgaa	121	0.51
39	6	78974466	gtagggcaagcggcaag	tgcttcctggacatttgga	110	0.14
40	4	68490768	ttgcttgggaacatggag	gcccttgtcatccactagga	121	0.00
41	1	41518626	cctggtgcaggttgcag	ccttcggagcccatgc	106	0.42
42	19	29399024	caggacacttgcaccagatt	gagcagaggtgaggctgaa	120	0.00
43	12	27463695	cagtagccaaattgtggaagc	accacgtagtcttgacccattc	68	0.30
44	16	10834862	gttcccttctcagaggacca	caatgagtgaagggggtcag	69	0.00
45	14	38539141	gagtggcacacgagcactt	gcaggactgtctggaggttg	68	0.31
46	8	60840667	tgcctgagggagctgtatatg	tctcattgtggagcaaagacat	75	0.51
47	5	36083663	gctttgttgtaagcagcgata	tgtgagaaactccattgcctta	74	0.21
48	7	53313567	agaaggggcagacttgagg	tccctcatttcacaagctga	75	0.19
49	1	88201810	tggctcattgatttgtgattct	ggccagctcttcttgttgag	78	0.00
50	14	61925547	gggttctctagggagatgacaa	aggacccaagtggattctga	83	Excluded
51	7	17212546	gtcatggtgacatcgcagtt	gcctcatgccaatgagagac	85	0.12
52	2	67041786	gatggccgattgtacatcaa	tggttgcagggaagattagg	95	0.34
53	5	3965764	ccgtctagttgtcgggtgtt	tgcagtatttagggtggagga	94	0.43
54	34	24388648	cccttgtaaaggggaggaga	tggctctgaatttaggcattt	94	0.48
55	12	23053279	agctctcctgctgtgattttt	tgcagacaaatggactgaaga	98	0.44
56	20	29687857	tgagcacgaagaggtagagaag	tcaagtgcaagtcaccaaact	100	0.43
57	30	37964459	cgtgaatggtccaaaatgat	catcagcatttccagagttctt	100	0.26
58	13	42973844	tttctgggcaaaaacagtga	tttagatgggagggaatggtt	108	Excluded
59	5	91665974	accttctgtttcccctttgg	agagcgcagcagagatgact	103	0.00
60	4	43098715	gtttacaccacccagcctga	aggcagttacaggcattaatca	109	0.40
61	20	40585796	cccatccctgaggaagagag	ggacacccgcatttctgtc	114	0.12
62	7	8798933	cccagaaaacaagagaaggaaa	tgttggcagatctcatggtc	114	0.00
63	14	23314695	acccaccagattggctaaaa	tcgaacaggtccagtttacatt	116	0.26
64	26	35769409	cctggcttaaccccttacct	aaccgctatcccacattctg	96	0.00
65	4	56495029	tcccactgtagcttgaaaacg	ttcaggtattgctgtccaaaaa	95	0.51
66	17	19537687	atgctttgcagagatgcttg	tgctcatgtcagaacagagagg	94	0.31
67	31	26928202	atgaacaagcaccccaaaac	cagtccacttcaatgcacca	68	0.27
68	10	66801300	ccccaaattaagggaagttca	tccatgaccaaatctgcatc	70	0.12
69	10	63275151	tccagaacttggagagaatcaa	tccaggcaagactatgagca	70	0.30
70	5	58727403	ctggaccacatatggcttga	tgccggagagatacgtgtaa	72	0.00
71	5	29583832	ccacacagtgttgcctgtagt	ggaaagcacagaaagttgtgaa	73	0.49
72	11	25068367	cccctgcttgtgctctctc	ctctcagcaacccacagagat	75	0.49
73	19	54304531	aagccttgcacttgagcttg	ccattggaaaggcacgtact	77	0.27
74	30	39573069	gcttttgtcatgaaacaccaa	ccaccagatgctcaagtctg	79	0.48
75	19	55236292	gcccacagggtctttattttt	gcttgggtctcttcctctctc	78	0.33
76	3	53761240	ctgtggtgcagcggtttag	ggagcctgacatgggactt	78	0.00
77	4	90768017	gttctccctgtgtctgactgtt	aaagaccaaggggtgaaaga	84	0.13
78	11	57430384	agggacagacccactaagtgtc	tccttaggcgacatggagac	86	Excluded
79	36	32128918	tgatatagccgaagtcaggaag	aatggaaagatcatccagacag	89	0.40
80	14	49479392	tttcaaattgctgaatgttgg	ccctggtctccaggatcac	89	0.47
81	27	13108059	cagccaattggacacaaaaa	aaaatcaagatgtcagcagagg	88	0.00
82	6	61627505	gtcatgatcccatggtccta	gtagaggggcagagggagag	88	0.00
83	36	14699428	gtgttttcttttgggcaagg	tgcaaccaacacacagatga	92	0.49
84	32	36108071	cccagtgttggtcacatataca	gcgatgaaaattgggaaaga	96	Excluded
85	17	57586353	gtgattagggttgaggggaga	ttttccatcaaggtttgtcca	97	0.42
86	5	16750608	aattttccaggaggctttgg	ttccctcctgctgatctagg	98	0.06
87	19	43306776	tggggtaaatcagtgagtgaag	ggcattaagagaagcctgctg	99	0.07
88	29	43945653	aacccgaataacattaggagga	tgggtttaagctggttacgg	104	0.51
89	5	31951980	cccagaaatccacttaatgacc	tgtgttaccagggctaggttc	104	0.44
90	7	50783779	ggttagcttagctcctctccaa	agccacatgctgaaaggaag	104	0.00
91	31	10957463	ttggcaactgccttacaataaa	ttgaatgtggacatgaaacaaa	106	0.47
92	16	6629437	gaaatgggaaggttttattcca	ggtgctgacaacagaaaacct	109	0.46
93	5	75136583	aggacacagacagatgtgagga	ccgaagaggaatctgcactc	111	0.00
94	3	89213750	gagaacttcgatgtgagggaat	ctctcccaccaaaaatctcct	113	0.29
95	21	51457175	aggcattcagggtgttaaaaa	tggtgaactggaaagtagctga	108	0.51
96	12	53379600	ttcccaaggagttggagaga	gctgagggcagctgtgttat	68	0.00
97	14	58723304	ccctgtggtaaccatcaacc	caaagtgaacaagcaaagcaa	67	0.51
98	3	11002853	ccactggccctaagtgactg	ttagggttttaaaggctgtgc	70	0.06
99	30	30015918	tcaggttttggatttgaagga	taagcacaaccattagctcca	72	0.06
100	7	65831405	gaggttcaaatttcccatatcc	gggatccatgcaaaatagttc	73	0.37

**Figure 1:**
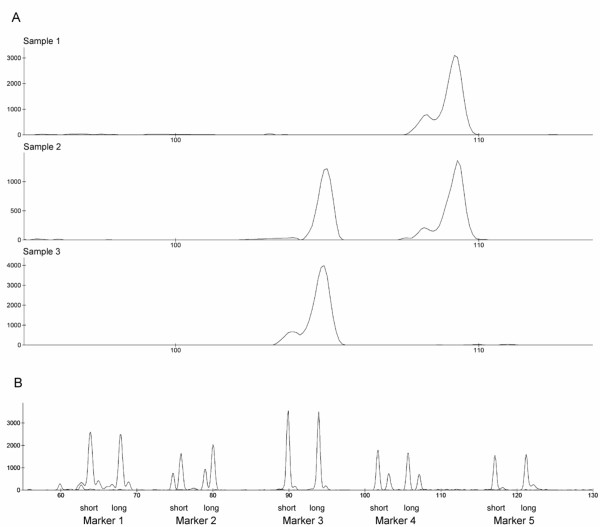
(a) Genotyping of a 4-bp indel locus in wolves showing (upper panel) a homozygote for the longer allele, (mid panel) a heterozygote and (lower panel) a homozygote for the shorter allele. (b) Multiplex amplification and simultaneous genotyping in a single capillary of five indel markers in one individual heterozygous for all these markers. The long and short alleles of marker 1–5 are labelled. All markers show some form of extra fragments that likely represent PCR artefacts. These may either be shorter (marker 2) or longer (marker 3–5) than the amplified allele, alternatively both shorter and longer (marker 1).

Using conventional genotyping based on fragment length separation in a DNA sequencing instrument, 81 out of the 94 putative markers were found to be polymorphic in a screening of 7 dogs and 76 of them were polymorphic in a global sample of 18 wolves (Figure 1A). As PCR primers were designed to generate amplicons of varying size within the 70-120-bp interval, combinations of multiplex reactions (three markers per PCR) were readily formed. This allowed simultaneous amplification, and consequently simultaneous genotyping within a single capillary, of several markers even using the same fluorofore (Figure 1B).

**Figure 2:**
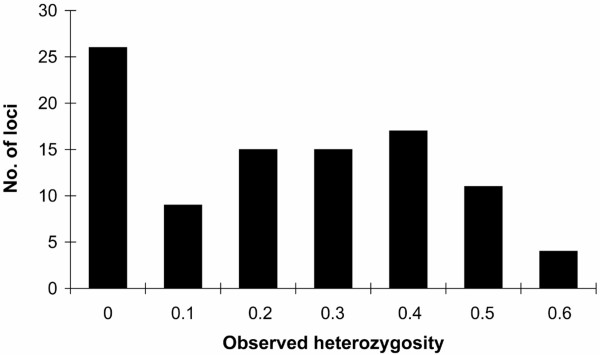
Distribution of observed heterosygosities at 94 indel loci genotyped in 18 wolves from five populations worldwide.

In wolves, 74% of the polymorphic loci had a minor allele frequency of >10% and 49% of >20%. The average observed and expected heterozygosities were respectively 19.4% and 26.1% in wolves, while they were 26.8% and 35.5% in dogs. The distribution of wolf heterozygosities is shown in Figure 2.

**Figure 3:**
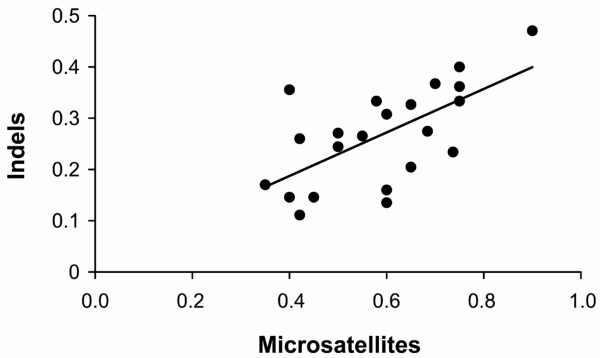
Correlation between average observed individual indel (51) and microsatellite (20) heterozygosities in 22 Swedish wolves.

The 76 indels found to be polymorphic in the global sample of wolves were subsequently genotyped in 27 wolves from a Swedish population. Fifty-one loci were polymorphic and showed an observed mean heterozygosity of 25.3%, or 17.0% if including all 76 markers. The same wolves were also genotyped for a set of 20 microsatellites known to be informative in this population [e.g. [21]]. Expected heterozygosities for these loci ranged between 28–75%. There was a positive correlation between mean heterozygosity at indel and microsatellite loci in individual wolves (*r*^2 ^= 0.41, *P *< 0.001; Figure 3).

## Discussion

Our study shows the feasibility of using large-scale genomic sequence data for extracting putative insertion and deletion polymorphisms, marker loci subsequently can be validated to represent informative genetic markers at a population level. It also demonstrates the feasibility of transfer of genomic data from a model species to a natural population of a close relative. Dogs were domesticated from wolves 10,000–100,000 years ago [22-24], and their divergence has since then been accentuated by strong artificial selection during domestication. Finally, by genotyping of indels and microsatellites in the same wolves it also shows that polymorphism levels of the two marker types are highly correlated.

A lack of large-scale genome sequence information has up till now hampered the introduction of indels as genetic markers in non-model species. It can be anticipated that this will come to change in the near future. There is a rapid increase in the number of genome sequencing initiatives and new sequencing technology, like "454-sequencing" [25], offers immense possibilities for generating massive amount of sequence data from hitherto uncharacterised genomes. Importantly, the depth of sequence coverage provided by new technology means that it is well suited for sequence analysis of pools of individuals, from which a wealth of polymorphism data can be obtained [26]. For example, if 100 Mb of sequence is generated from each of two individuals (with a 1 Gb-genome) in two mega-sequencing runs, and with an indel density of 1 every 2 kb in pairwise comparisons, several hundred indels are expected to be detected.

Indel density has not been as well characterized in natural populations as nucleotide diversity. In domestic chicken, the pairwise heterozygosity for indels is 2 × 10^-4 ^per bp [15]. In a natural population of collared flycatchers, Backström et al. [27] found a similar occurrence of indels, 1–2 × 10^-4 ^per bp. In this study we found about 30,000 indels in 7.2 Mbp of dog sequence, which translates into a heterozygosity of 4 × 10^-4 ^per bp. This includes length variants in unique sequence as well as in repetititve DNA, like microsatellites. Using a similar search algorithm and a similar type of shot-gun vs. genomic reference data set for chicken, we recently found that about half of all length variants detected in this way represent tandem repeats [15]. This would suggest that in dogs, the heterozygosity for short non-repetitive indels is about 2 × 10^-4 ^per bp, similar to chicken. Moreover, the length distribution of dog indels (Table 1) show congruence with such data from chicken.

The Swedish wolf population was functionally extinct by the 1960s–1970s but has subsequently recovered to a current size of well over 100 individuals [28]. All contemporary Scandinavian wolves are thought to originate from only three founders, that were eastern immigrants arriving to Sweden around 1980 and 1990, respectively [21]. The strong bottleneck, subsequent inbreeding and the associated loss of genetic diversity experienced by this population [21,29], give the opportunity to test the utility of indel markers in a small and endangered natural population. The finding that about 50% of *in silico *predicted indels from pairwise sequence comparisons of dog alleles is informative in this wolf population confirms the usefulness of indel markers even in a population with limited genetic diversity.

The mean heterozygosity of the 51 polymorphic indels within the Scandinavian wolf population (25%) is somewhat lower than what was been observed for 21 SNPs (34%) in the same population [29]. However, those SNPs were initially identified from a screening of a limited number of Scandinavian wolves so there was an ascertainment bias in favour of markers with high polymorphism information content. Generally, for those indels and SNPs that represent neutral markers, there should be no reason to believe that heterozygosity for polymorphic loci differs between the two marker categories. Indels in coding sequence are likely to more often be deleterious than point mutations, at least indels that cause frame shift mutations, which should act as to reduce their diversity due to negative selection. On the other hand, point mutations in coding sequence may potentially more often than indels be subject to positive selection, which also reduces diversity. In any case, although probably comparable to SNPs, indels do show less variation than microsatellites. Thus, to obtain the same resolution power in relatedness analyses, a higher number of biallelic markers are needed compared to multiallelic microsatellites [30-32]. However, the rich abundance of indels in genome sequence surveys and the ease by which they are genotyped (Figure 1a) and multiplexed (Figure 1b) add to their benefit. Moreover, it is possible to design microarrays specifically for short indels, by which genotyping costs become very low [32].

## Conclusion

With an increasing amount of sequence information gathered from non-model organisms, we suggest that indels will come to form an important source of genetic markers, easy and cheap to genotype, for studies of natural populations.

## Methods

### Samples

Genomic DNA was extracted from wolf tissue samples using standard phenol-chloroform extraction protocols or the DNEasy Tissue Kit (Qiagen). Altogether 18 samples, from Sweden (5), Finland (3), Spain (3), Russia (2) and Canada (5), were used to test the amplification ability and to get a first idea of polymorphism of indels. Seven domestic dog samples from different breeds (Dachshund, Dalmatian, Gordon Setter, Greenland Dog, Lakeland Terrier, Pyrenean Mountain Dog, Welsh Corgi) were also added. Subsequently, we tested the ability of indel markers to analyse the genetic diversity at the intra-populational level using tissue samples of 27 wolves collected between 1985 and 2005 from roadkills or shot animals from Sweden [21,29].

### Selection of markers

A total of about 200,000 dog trace read sequences were obtained from GenBank. These sequence tags were almost exclusively derived from light shot-gun sequencing of unrelated dogs that was done in conjunction to the sequencing of the dog genome [18]. An automated pipeline was set up to survey the sequences for potential indels and for design of primers. The initial step in the pipeline was to place all STS sequences onto the dog genome. This was done using local NCBI BLAST [33], with a conservative setting to require an E value of less than 10^-70^. To avoid possible duplicated loci all cases where there was more than one BLAST hit were discarded. Next, the BLAST results were surveyed for 4 bp indels, recognised as 4 bp gaps in alignments of shot-gun reads and the genome reference sequence. To avoid selection of microsatellites only those 4 bp indels where none of the flanks were identical to the indel were used for further processing. For each indel with at least 70 bp flanking sequence on both sides, Primer3 [34] was used for primer design. Primers were requested from the program for fragment lengths between 70 and 120 bp. The primers were constrained by a required melting temperature between 58 and 62°C, as well as a primer length between 19 and 22 bp. Finally the primers were evaluated with regard to self complementarity, as well as for the possibility of the resulting product to form a hair-pin. This was done through a simple complementarity testing procedure where possible self-complementarity at the sharp end of the hairpin was scored higher, and decreasing score inwards. The top 100 loci passing through all steps were picked for screening. Primers were fluorescently labelled with either FAM, HEX, or TET.

The same animals were also genotyped for a set of 20 autosomal microsatellites, as described in ref. 20: c2001, c2006, c2010, c2017, c2054, c2079, c2088 and c2096, vWF, u109, u173, u225, u250 and u253 and PEZ01, PEZ03, PEZ05, PEZ06, PEZ08 and PEZ12.

### Genotyping and data analysis

Amplification by polymerase chain reaction (PCR) was performed in 10 μl solution containing 20 ng DNA, 0.25 U AmpliTaq Gold polymerase with 1× Amplitaq Gold PCR buffer (Applied Biosystems), 2.5 mM MgCl_2_, 0.3 μM of each primer and 0.4 mM dNTP. The PCR profile for the indel markers included initial heating at 95°C for 5 min, followed by 35 cycles of 95°C for 30 s, 58°C for 30 s and 72°C for 1 min, and a final extension at 72°C for 10 min. The profile for microsatellites included an initial denaturation step of 95°C for 10 min, 11 touch-down cycles with 94°C for 30 s, 58°C for 30 s, decreasing by 0.5°C in each cycle, and 72°C for 1 min, then 28 cycles of 94°C for 30 s, 52°C for 30 s and 72°C for 1 min and a final extension of 72°C for 10 min. PCR products were run on a MegaBACE 1000 capillary sequencer (Amersham Biosciences) and analyzed using the accompanied software Genetic Profiler 2.2. Observed and expected heterosygosities calculated using Microsatellite Toolkit for MS Excel [35], and correlation between the observed individual heterozygosities according to indel and microsatellite data was estimated.

## Authors' contributions

ÜV carried out the molecular studies and performed the data analysis. MB participated in the design of the study, selected markers and designed primers. MJ participated in the molecular analyses. HE conceived of and coordinated the study, and wrote the paper together with ÜV. All authors read and approved the final manuscript.
